# Laser Capture Micro-dissection (LCM) of Neonatal Mouse Forebrain for RNA Isolation

**DOI:** 10.21769/BioProtoc.3475

**Published:** 2020-01-05

**Authors:** Achira Roy, Mei Deng, Kimberly A. Aldinger, Ian A. Glass, Kathleen J. Millen

**Affiliations:** 1 Center for Integrative Brain Research, Seattle Children’s Research Institute, Seattle, United States; 2 Division of Genetic Medicine, Department of Pediatrics, University of Washington, Seattle, United States

**Keywords:** Mouse, Forebrain, Laser capture microdissection, RNA isolation, Neural progenitors, Microscopy, RNA sequencing, Hippocampus

## Abstract

Precise and reproducible isolation of desired cell types or layers from heterogeneous tissues is crucial to analyze specific gene profiles and molecular interactions *in vivo*. Forebrain is the core site of higher functions, like cognition and memory consolidation. It is composed of heterogeneous and distinct cell types, interconnected to form functional neural circuits. Any alteration in the development or function often leads to brain disorders with profound consequences. Thus, precise molecular understanding of forebrain development in normal and diseased scenarios is important. For quantitative studies, most traditional analytical methods require pooling of large cell populations, that results in loss of *in vivo* tissue integrity and of spatial, molecular and cellular resolution. Laser capture microdissection (LCM) is a fast and extremely precise method of obtaining uncontaminated, homogeneous sets of specific cell types and layers. Our current procedure involves cryo-sectioning and laser microdissection of fresh-frozen mouse forebrains, that are genetically modified and treated with small-molecule therapeutics. Using LCM, specific regions of interest, such as neural layers, cells from adjacent yet distinct subregions within a tissue layer, are obtained under RNase-free conditions. These small cellular cohorts are further used for downstream, high-throughput genomic or transcriptomic assays. Here, we have introduced break-points at multiple stages throughout our protocol. This makes our method simpler and more user-friendly to follow, without compromising on the quality. The current protocol can easily be adapted for different brain regions, as well as for other model organisms/human tissue.

## Background

To understand the molecular mechanisms underlying normal and diseased neural processes, it is critical to analyze the interactions *in vivo*, within a specific tissue microenvironment. It is quite challenging to precisely access different areas of the developing brain, especially in deep areas like hippocampus. The level of complication increases further due to the existence of cellular heterogeneity even within a single neural layer. Traditional quantitative methods like microarray *etc.*, using gross tissue lysates or various primary and organotypic culture systems, rely heavily on pooling gene expression data from thousands of cells and reporting a population-wide average (Levsky and Singer, 2003). Thus, these procedures fail to capture subtle differences within distinct cell subsets as well as to maintain *in vivo* tissue/cell integrity. Moreover, there remains a high risk of tissue contamination from neighboring areas during sample collection. Laser-capture microdissection (LCM) combines the benefits of light microscopy and precision of laser beam to procure homogeneous subpopulations of cells from heterogeneous tissue under direct microscopic visualization (Emmert-Buck *et al*., 1996). The laser cutting is usually less than 1 μm wide. This allows target cells to remain unaffected by the laser beam. LCM technology can selectively and routinely harvest regions of interest, ranging from specific tissue layers to single cells and chromosomes (https://www.leica-microsystems.com/products/light-microscopes/details/product/leica-lmd7/). This enables the user to obtain reproducible, targeted starting material for biologically relevant data analyses.

Data using the described LCM protocol was published in (Roy *et al*., 2019). In this study, we found that activating mutations in the phosphoinositide 3-kinase (PI3K) pathway results in focal disruption of cell-cell adhesion, proliferation and apical tissue integrity, leading to cortical gyrification (in both neocortex and hippocampus) and developmental hydrocephalus in mice. Conventional quantitative analytical methods were not precise enough to distinguish cellular/molecular heterogeneity within a neural layer, between adjacent gyral and sulcal zones in the mutant mouse brain. Hence, we used LCM to specifically dissect selective portions of the developing ventricular zone from P0 control and mutant littermates, followed by RNA sequencing. This data was then analyzed to study the effect of the *PI3K* mutation on neural progenitors. Further, we performed the same experiment after administering a small-molecule drug *in utero*, to compare the effect of drug treatment on the gene expression profile between controls and mutants.

Here, we present detailed step-wise instructions including experimental ‘do’s and don’ts’, and also illustrate key components and steps using photographs, to aid new researchers in setting up this useful procedure. We have introduced a number of stopping steps throughout the protocol. This makes our method simple and more user-friendly, without making any compromise on the quality, quantity and integrity of the end product (RNA, DNA *etc.*). This method can be used with fresh-frozen and fixed tissues.

Some other applications of this protocol are as follows:

1. Absolute identification of possible mosaic mutations with cells of interest, in mouse models or human patients.
2. Downstream analyses like selective quantitative Real-Time PCR, protein assays like Western blot analysis, chromatin immuno-precipitation, genomics (DNA assays), as well as metabolomics.
3. Live cell culture, cloning and genetic manipulations.
4. Isolation of small quantity of specific tissue material, highly useful for plant biology, forensic science and prenatal diagnostics.

## Materials and Reagents

1. Sterile gloves
2. DNase-free, RNase-free micropipette tips
3. Fisherbrand Permanent fine tip marking pen (Fisher Scientific, catalog number: 13–379-4), to label embedding molds, and tubes for genotyping, if required
4. Fisherbrand Petri Dish with Clear Lid 100 mm x 15 mm, sterile (Fisher Scientific, catalog number: FB0875713)
5. Bucket of Ice
6. Embedding molds (Thermo Scientific, catalog number: 70182)
7. Cryogenic Fiberboard Freezer Box, 2’’ (VWR International, catalog number: 89214–750)
8. Regular duty single edge blades (Personna, catalog number: 94–115-71)
9. Low Profile Microtome Blades (Sakura, Accu-Edge, catalog number: 4689)
10. Fisherbrand Superfrost Plus microscope slides, precleaned (Fisher Scientific, catalog number: 12–550-15)
11. MembraneSlides, nuclease and human nucleic acid-free polyethylene naphthalate (PEN)-Membrane 2 μm (PEN slides) (Leica Microsystems, catalog number: 11505189)
12. Pencil, to label slides
13. New plastic 5-slide mailer–open top (Electron Microscopy Sciences, catalog number: 71548–01), for storage of PEN slides
14. Sterile 50 ml Polypropylene conical tube (Falcon, catalog number: 352070) for staining or storage of PEN slides
15. Kimwipes (Kimberly-Clark Professional, catalog number: 34155 or 34120)
16. Samco™ Graduated Transfer Pipettes, sterile individually wrapped (Thermo Fisher Scientific, catalog number: 274–1S)
17. Tissue collection tubes: 0.5 ml Tubes with Flat Cap, certified DNase/RNase-free (Thermowell Tubes, catalog number: 6530)
18. High quality bags from VacUpack (for example: 4 Mil Vacuum Bags, size: 8”X10”, U.S. Vacuum Packaging and Wrapping LLC., catalog number: SS-VB4-0810)
19. Small biohazard bags, to dispose of mouse carcasses (Medline, catalog number: DYND30261)
20. RNase-free 1.7 ml tubes (Olympus Plastics, Genesee Scientific, catalog number: 24-282A)
21. Aqui-Pad Laboratory Mat (Therapak, catalog number: 10324G)
22. **Animal:** Mouse, neonatal pups (P0-P6)
  Notes:
  - Male and female mice are mated, and embryos are staged considering the morning a vaginal plug is detected as embryonic day (E)0.5. In utero drug treatment can be done using this staging. Specifically, the types of mating used in (Roy et al., 2019) are described in detail in the article’s Methods section.
  - The day the pups are born is considered as P0. For FVB strain, the gestation period is E18-E19.
  - No alterations to the protocol are required if different mouse strains are used.
23. RNasin Plus RNase Inhibitor (Promega, catalog number: 5067–1513)
24. *Desiccants:* Indicator blue Drierite™ desiccant (W.A. Hammond Drierite Company Ltd., mesh size: 8, stock number: 23005); regular Drierite™ desiccant (W.A. Hammond Drierite Company Ltd., mesh size: 8, stock number: 12001)
25. RNaseZap RNase Decontamination Solution (Invitrogen by Thermo Fisher Scientific, catalog number: AM9780)
26. Phosphate Buffer Saline PBS pH 7.4 (1x) (Gibco, catalog number: 10010–023)
27. Dry Ice
28. Optimum Cutting Temperature (OCT) Embedding Compound (Electron Microscopy Sciences, TissueTek #4583, catalog number: 62550–01)
29. Cresyl violet powder (Sigma-Aldrich, Harleco, catalog number: 190–12), storage: +15 °C to +30 °C
  Note: Keep the bottle RNase-free.
30. RNeasy Plus Micro Kit (50) (Qiagen, catalog number: 74034, storage: RNeasy MinElute Spin Columns at 2–8 °C, remaining components at room temperature (15–25 °C)
31. Agilent RNA 6000 Pico Kit (Agilent Technologies, catalog number: 5067–1513), storage: 4 °C, RNA ladder at −80 °C
32. Molecular Biology Grade Water (Corning, catalog number: 46–000-CM)
33. Ethanol 200 Proof Anhydrous (Decon Labs, Inc., catalog number: 2716)
34. 2-Mercaptoethanol (β-mercaptoethanol) (Bio-Rad, catalog number: 161–0710)
35. 70% ethanol (100 ml) (see Recipes)
36. 95% ethanol (100 ml) (see Recipes)
37. RNaseZap/70% ethanol mixture (100 ml) (see Recipes)
38. 75% ethanol (freshly-prepared) (see Recipes)
39. Nissl stain for RNA research (50 ml) (see Recipes)

Note: If the experiment requires comparison of mouse tissue between control and genetically modified mutant models and/or ±drug treatment, reagents to perform mouse tissue genotyping are required as briefly mentioned in (Roy et al., 2015 and 2019).

## Equipment

1. Clean micropipettes
2. 16oz Rubber Mallet with Wood Handle, to crush dry ice (Olympia Tools, catalog number: 60661116)
3. Dissection instruments:
  - Dumont fine forceps #5 (Fine Science Tools, catalog number: 11252–20)
  - Dumont fine forceps #55 (Fine Science Tools, catalog number: 11255–20)
  - Dumont coarse forceps AA (Fine Science Tools, catalog number: 11210–20)
  - Fine Scissors–Sharp (Fine Science Tools, catalog number: 14060–10)
4. Fisherbrand™ Stainless Steel Micro Chattaway Spatulas (Fisher Scientific, catalog numbers: 11543482 and 14–373)
5. Autoclave
6. Cryostat (Leica Microsystems, model: CM1850)
7. Brightfield Leica EZ4 Stereo Microscope (Leica Microsystems, catalog number: 10447197)
−80 °C freezer
−20 °C freezer
10. 4 °C refrigerator
11. Polyethylene ethanol wide-mouth wash bottle with polypropylene cap; 500 ml (Bel-Art SP Scienceware, catalog number: F11816–0019)
12. Laser Microdissection Microscope (Leica Microsystems, model: LMD7)
13. Agilent 2100 Bioanalyzer (Agilent Technologies, catalog number: G2939BA)
14. Chip priming station (Agilent Technologies, catalog number: 5065–4401)
15. IKA MS3 vortex mixer for Agilent 2100 Bioanalyzer
16. NanoDrop 2000 microvolume spectrophotometer (Thermo Scientific, catalog number: ND-2000)
17. Vacuum Desiccator (Fisher Scientific, catalog number: 08–648-107)
  Optional: Desiccator, home-made using stacked mixture of regular Drierite and Indicator blue Drierite desiccant, in a large glass bottle.
18. Microcentrifuge (Eppendorf, catalog number: 5417R)
19. Vortex (Scientific Industries, model: Vortex Genie-2, catalog number: SI-T236)
20. Analytical electronic balance (Reshy, catalog number: JM-B)
21. UV transilluminator (one used here is from Fotodyne, catalog number: FD33500; should preferably be used only RNase-free and without any chemical contamination)
22. Rocking shaker (Reliable Scientific Inc., model 55), for solution preparation and staining of slides.
23. Vacuum Sealing System with Starter Kit (Foodsaver, catalog number: V2244)

Note: If the experiment requires mouse tissue genotyping, appropriate set of equipment should be available.

## Software

1. Laser Microdissection System (Leica Microsystems, version: 8.2.0.6739)
2. Agilent 2100 Expert Software (Agilent Technologies, version: B.02.08.SI648(SR2))
3. NanoDrop software (Thermo Scientific, version used: 1.6.198)
4. *Optional:* Microsoft PowerPoint

Note: For genotyping, Image Lab™ Software (Bio-Rad, 1708265) is required for gel documentation.

## Procedure

Note: Prior to starting laser microdissection, the user should be well trained in cryo-sectioning and have sufficient knowledge of microscopy and histopathology to sample the correct tissue or cells. To minimize quality damage, the whole procedure of tissue handling should be carried out quickly, yet carefully.

### A. Preparation of solutions and set-up for brain dissection

**Figure 1.**
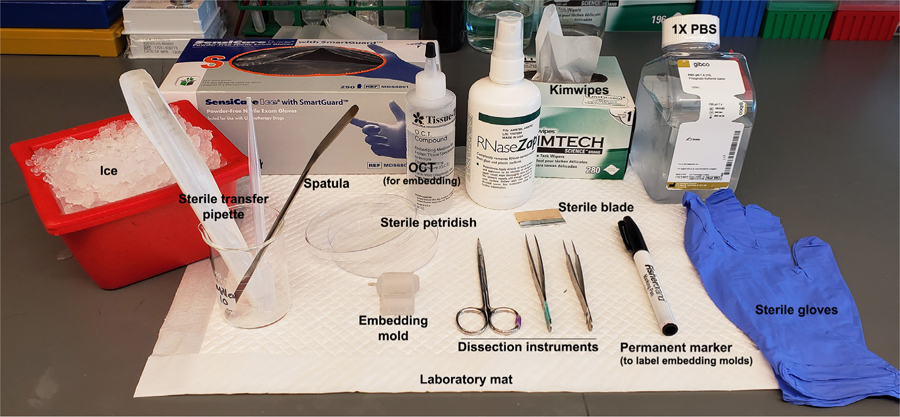
Materials needed for dissection of neonatal mouse forebrain. Reagents and equipment needed for mouse brain dissection are labeled. 1x PBS–Phosphate buffer saline, working solution.

**Figure 2.**
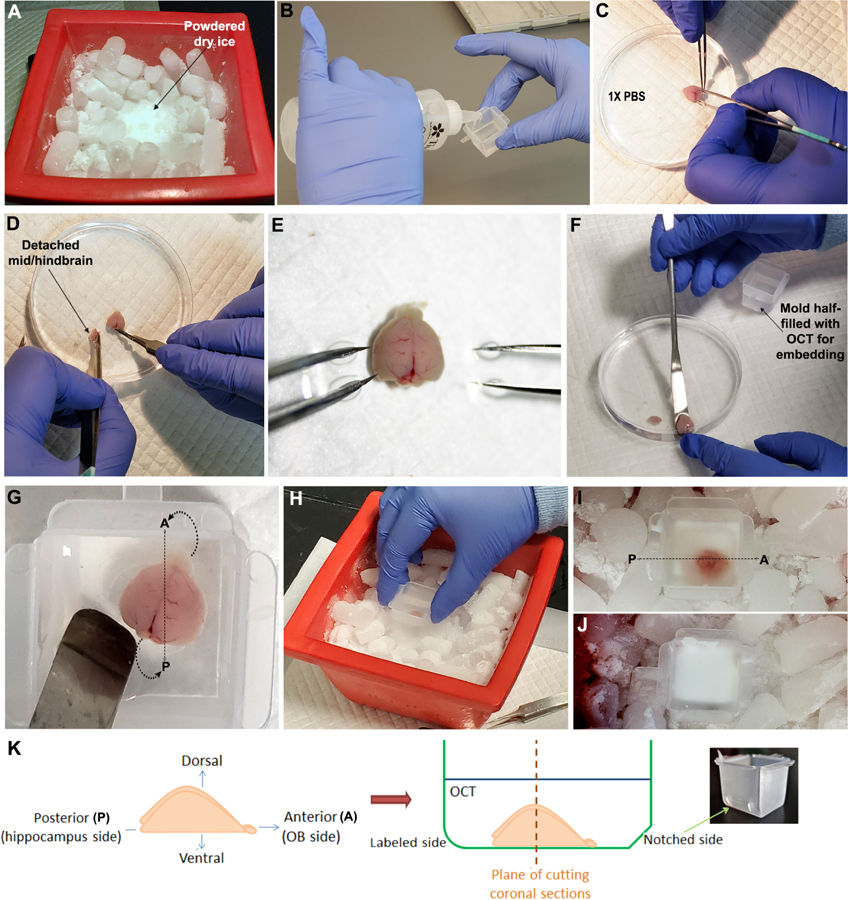
Steps of dissecting and embedding a neonatal mouse forebrain. A. Crushed dry ice prepared for freezing the embedding mold. B. OCT poured along an edge of the embedding mold to avoid air bubbles. C-E. Dissection of neonatal mouse forebrain, in sterile 1x PBS. F-G. Forebrain carefully transferred to and then oriented in the embedding mold filled with OCT. H-J. Mold with OCT-covered oriented mouse forebrain placed in dry ice, until OCT freezes to opaque form. K. Schematic of how forebrain was oriented, to be used for coronal sectioning (Roy *et al*., 2019).

Notes:

- Wear sterile disposable gloves and change frequently at every step, from preparing reagents till RNA analysis etc.
- Use clean, RNase-free instruments. Before handling the next specimen, spray the instruments with RNaseZap, rinse with RNase-free water and wipe with clean Kimwipes.
- Use RNase-free or Nuclease-free solutions, glassware and plastic ware.
- Use RNaseZap or similar product to clean the equipment or the surface of the working area.
- Freeze tissues immediately after surgery to obtain high-quality RNA. This minimizes gradual RNA degradation by ubiquitous RNases or heat.

1. Prepare working solutions, as mentioned in the Recipes section
2. Label the cryomolds at room temperature using the Fisherbrand permanent marker, prior to the tissue dissection experiment, preferably on the side-wall opposite to the notched side (for easier orientation)
3. Autoclave the dissection instruments and spatula for sterilization, prior to the experiment.
  Note: The dissection tools should have never seen any fixative etc.; wipe them with RNaseZap, then RNase-free DNase-free water and dry with clean Kimwipes before starting the experiment.
4. Wipe the tissue dissection area thoroughly with 100% RNaseZAP solution, let the area dry before placing the laboratory mat for dissection.
5. Assemble all reagents and equipment before tissue dissection, as shown in Figure 1.
6. Crush some dry-ice using the mallet to generate a soft powdered area for future placement of the embedding mold (Figure 2A).
7. Pour OCT slowly and carefully along one edge of the sterile embedding mold till it is half-filled (Figures 2B and 2F).
  Note: Avoid air bubbles in the OCT while pouring. Air bubbles disrupt the tissue integrity.

### B. Neonatal mouse brain dissection

1. Euthanize neonatal mouse pups of the desired age (P0-P6) using procedures permitted by your Institutional guidelines. In Roy *et al*. (2019), as per the approved animal protocol (see Ethics statement), euthanasia was performed by inducing hypothermia anesthesia, followed by decapitation. For this procedure, place the newborn pups on the lid of a Petri dish and set on top of crushed ice for 2–3 min, leading to their unconsciousness. Confirm anesthesia in pups by lack of response to firm toe pinch, once they stop moving. Decapitate using sharp scissors.
2. Transfer the heads to a sterile 100 mm Petri dish containing sterile 1x PBS.
3. At this stage, if genotyping of the pups is required, take a tiny piece of the tail (≤ 2 mm) of each pup and place them in labeled 1.7 ml microcentrifuge tubes for further processing.
4. Dispose of the carcasses using labeled biohazard bags.
5. Under a dissection microscope, dissect out the brains using fine and coarse forceps (Figure 2C). Dispose of the remaining carcasses using the biohazard bags.
  Note: If the Petri dish becomes murky with blood, you can transfer the brains at this stage to another Petri dish containing sterile 1x PBS.
6. After removing the brain, sever the hindbrain and midbrain, retaining only the forebrain including the olfactory bulbs (Figures 2D–2E). The presence of the olfactory bulb helps to position the telencephalic hemisphere in the correct orientation in the embedding molds.

### C. RNase-free brain mounting

1. With the help of the spatula and coarse forceps (if needed), take the brain out of the buffer and transfer to the pre-labeled embedding mold half-filled with OCT (Figures 2F–2G).
  Note: Try to remove liquid from the brain surface as much as possible without damaging the brain (you may carefully use a finely rolled Kimwipe if needed), before immersing it in the OCT.
2. Orient the mouse forebrain such that it is parallel to or on the floor of the embedding mold.
3. For coronal sectioning (perpendicular to the antero-posterior axis of the forebrain), slowly orient the anterior side of the brain (olfactory bulb side) directly perpendicular to the notched side of the mold. Avoid air bubbles in the process (Figures 2G and 2K).
4. Once the brain is properly oriented, slowly pour OCT along one edge to fill the mold (or at least enough to cover the entire brain). Take precaution that the brain orientation remains intact and air bubbles do not develop in between OCT layers.
5. Carefully place the mold within the powdered dry ice and support the mold from the sides by pieces of dry ice (Figures 2H–2I).
6. Once the mold, with brain embedded, becomes completely opaque (Figure 2J), transfer it to a new, clean freezer box and immediately store at −80 °C freezer.

### D. Preparation before tissue sectioning

1. Clean cryostat, microtome blade and tools with a 1:1 mixture of RNaseZap and 70% ethanol (RNaseZap/70% ethanol) and then with sterile 95% ethanol (Recipes 1-3). Wipe dry with Kimwipes.
2. Incubate PEN membrane slides for 30 min under UV light (on the UV transilluminator) to enhance the binding of the tissue to the membrane slide.
3. Get a bucket of dry ice for tissue/embedded mold transportation.
4. Prepare solutions with RNase-free water and high-quality ethanol:
  - 75% ethanol (freshly prepared, Recipe 4)
  - Nissl stain (Recipe 5, prepare at least 3 days before use and store at 4 °C). Aliquot the amount needed for current use, by roughly estimating the total number of slides to be stained (10–15 drops of the solution is adequate to rapid stain each slide).
5. Set temperature of cryostat in the range of −15 °C to −18 °C
6. Label PEN slides with brain code/genotype using a pencil.

### E. Cryo-sectioning of brain tissue

**Figure 3.**
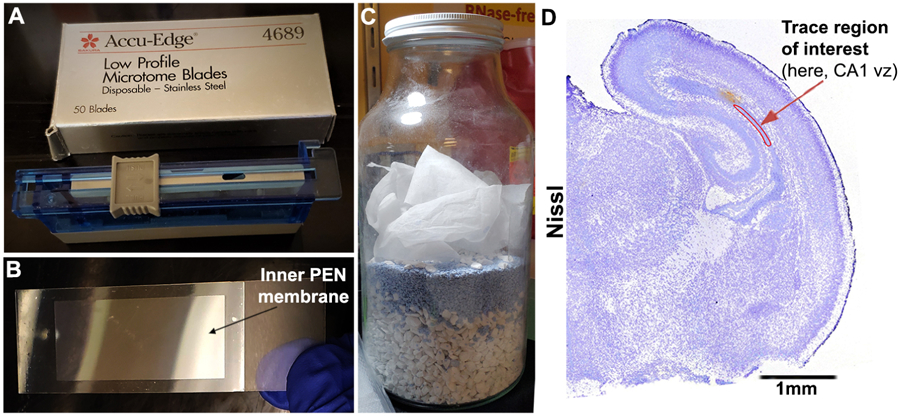
Preparation for tissue sectioning and laser microdissection. A. New sterile microtome blades used for brain sectioning. B. PEN slides: mount sections onto the inner film, which finally gets laser-cut. C. Home-made desiccator using Drierite to dry slides prior to LCM. D. Representative image of a Nissl-stained coronal hemi-section of P0 mouse forebrain, used in microdissection and data analysis (Roy *et al*., 2019), showing how region of interest is traced. In this context, ventricular zone of P0 mouse hippocampal CA1 region (CA1 vz) was traced.

1. Wear new sterile disposable RNase-free gloves. Change gloves frequently between steps to maintain the RNase-free status.
2. Transfer an embedded brain mold, placed securely on dry ice, from −80 °C freezer to the cryostat.
3. Use new sterile, single-edge blade to tear the plastic embedding mold inside the cryostat. Trim the OCT-embedded block as needed.
4. For coronal staining, attach the block on the cryostat chuck (specimen clamp) such that the anterior side of the brain (notched side of mold, Figure 2K) faces perpendicular to the microtome blade inside the cryostat.
5. Place embedded brain inside the cryostat at least 20 min prior to sectioning, in order to allow it to come to the cryostat temperature.
6. Use a new sterile microtome blade for each experiment (Figure 3A).
7. Slice a few sections from the surface of the mold to avoid any sort of contamination, prior to the collection of actual sections to be mounted on slides.
8. Cut 8–10 μm sections and mount on regular charged plus slides (on the positively-charged coated side). To identify your region of interest, rapid stain with Nissl staining solution or other dye for about 1 min, swivel gently, and observe slides under stereo microscope.
  Note: Staining procedure is explained in Step E14b.
9. Once you start getting sections of your region of interest, change the slicing thickness to 16–18 μm and mount on the PEN membrane slides. You can mount multiple sections on one slide. For example, ~12–14 sections were mounted on each PEN slide in Roy *et al*. (2019).
  Note: Mount the sections within the extent on the PEN-coated membrane (Figure 3B). Please take care that the coating does not get any scratches or leave the RNase-free area.
10. Optimally, place the PEN slides on the desiccant during the section collection.
11. Clean the blade and cryo-sectioning stage with brush or Kimwipe in between sections.
12. Collect one section of every 5–10 sections and mount on the regular charged slide for histologic staining.
13. Air-dry the PEN slides at room temperature for about 2 min on desiccant. Make sure the collected sections are completely dry before staining.
14. After air-drying, PEN slides with collected sections can be:
  - Stored in plastic slide mailer in a vacuumed bag, containing desiccant, at −80 °C, for a few months or longer, until user is ready to perform LCM.
  - Immediately transferred to 100% ethanol at room temperature for dehydration, followed by Nissl staining.
    The Nissl staining protocol is as follows [a quicker staining method, modified from (Grundemann et al., 2008) and Leica LMD Protocol Guide (https://www.leica-microsystems.com/products/light-microscopes/details/product/leica-lmd7/)]:
    - Stain slides one at a time using 50 ml tube or simultaneously using a big container.
    - Immerse the slides in fresh 100% ethanol, 5 min x 4 times, and place on the rocking shaker to remove any OCT attached.
    - Immerse slides in freshly-prepared 75% ethanol (Recipe 4) for ~1 min.
    - Place drops of Nissl stain (Recipe 5) directly onto the sections, using plastic transfer pipette, and incubate for 1 min. Swivel the slides gently. On average, 10–15 drops of the solution are adequate per slide.
    - Quickly rinse the slides with fresh 100% ethanol, 5 s x 3 times.
    - Finally, immerse the slides in fresh 100% ethanol for 1 min, to allow the stain to bind onto the sections.
    - Observe sections under a brightfield dissecting microscope.
    - Allow the slides to stand for at least 30 min in order to make them completely dry before storage or use.
    *Note:* Optimally, finish staining of sections strictly within 10 min in total.
15. Store completely dried PEN slides with mounted sections at −80 °C, either before or after Nissl staining. Neither procedure will negatively affect the RNA quality. Keep the slides at −80 °C until you are ready to start LCM.

### F. Laser microdissection

**Figure 4.**
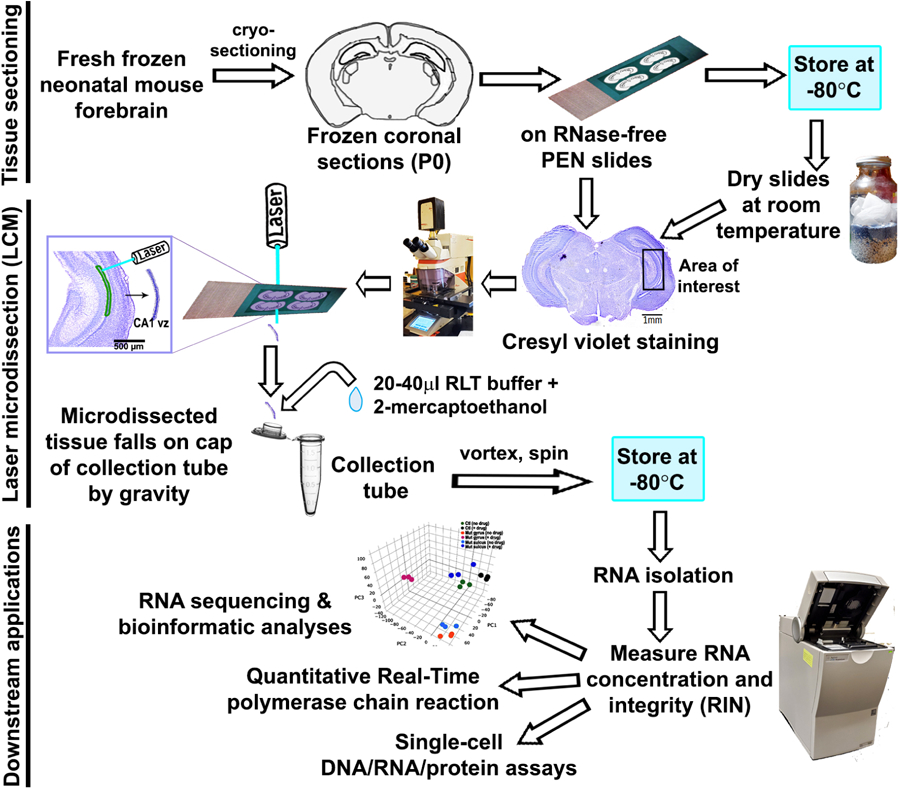
Flowchart showing different steps of laser microdissection of neonatal mouse forebrain tissue. Different steps–tissue sectioning, laser-capture microdissection, downstream applications. Some images are obtained from Roy *et al*. (2019), where we compared the genetic profiles of the hippocampal CA1 ventricular zone (CA1 vz) between P0 control and activating *Pik3ca* mutant ± drug, using RNA sequencing.

Notes:

1. All steps of laser microdissection should be performed in a designated RNase-free zone, in a clean, steady air-conditioned room.
2. Be cautious to slowly adjust frozen slides to room temperature before laser microdissection. This is critical for RNA quality since water (moisture) activates RNases.
3. Wipe the work area with Kimwipes dipped in RNase-free water, followed by 100% ethanol.
4. Clean the stage, substage and UV shield of the LMD machine sequentially with 95% and 100% ethanol.

1. Take the PEN slides (in the vacuumed bag) out of −80 °C freezer and allow it to slowly reach room temperature, for at least 60 min, before opening the bag. This is to avoid formation of water condensation on the sections. This step is critical for preserving RNA quality. Another method of drying the slide is to use the vacuum desiccator or a home-made RNase-free desiccator (Figure 3C) and dry the slides on the same day.
2. Switch the power of the Leica LMD equipment on.
3. Clean the collect tube caps (0.5 ml) with RNaseZap, followed by a rinse with RNase-free water.
4. Stain the section collected on regular slide (see Step E12) to confirm the area of interest, before performing LCM on serial sections mounted on PEN slides.
  Alternate method: If one studying the scientific question and one executing the laser microdissection are separate personnel, working at separate places/time zones, a crude method to designate the region of interest can be followed:
  - Image the Nissl-stained forebrain sections mounted on the regular plus slides, 1 for every 10 sections mounted on PEN slides.
  - Copy and paste each image as a separate slide in Microsoft PowerPoint.
  - Carefully trace the region of interest, using PowerPoint tools, as shown in Figure 3D.
  - Using these representative tracings, successfully perform LCM on the related PEN slides having the respective serial sections.
5. If not already done, stain sections on the PEN slides with Nissl stain, as described in the previous section, just prior to microdissection.
6. Dry the slides post-staining, in the desiccator.
7. Place PEN slides upside down in the stack-slide holder.
8. Create a sample overview under 1.25x lens, using LED/halogen illumination.
9. Mount 0.5 ml RNase-free, DNase-free collection tubes in the LMD tube holder. Add 20–40 μl of a mixture of RLT buffer and 2-mercaptoethanol to the cap of each tube (10 μl 2-mercaptoethanol in 1 ml RLT buffer from Qiagen RNeasy Plus Micro Kit).
10. Set the LMD software to Transmitted light-brightfield (TL-BF) mode.
11. Adjust software parameters to get suitable cutting including more accurate and less damage of tissue. Software parameters used in Roy *et al.* (2019): Draw shapes using “close line”. Use the “Move + Cut” mode for cutting respected tissue zones/cells. Use “Fine” X-Y-Z precision mode.
12. Guide the laser beam with mouse or touchscreen over your specific region of interest. The dissected tissue drops into the caps of collection tube by force of gravity without contamination. In Roy *et al*. (2019), ventricular zone of P0 hippocampal CA1 field (CA1 vz) was collected from each coronal hemisphere (Figure 4).
  Note: Please load a new collection tube every 2 h if longer cutting time needed.
13. Once all sections of the first slide are laser-cut, move to the next slide and repeat Steps F9-F12. You can store the used slides for future inspection and documentation.
  Note: Depending on the size and density of the laser-dissected samples, you can decide whether to pool together samples obtained from each brain for RNA isolation and further processing. In Roy et al. (2019), our region of interest (ventricular zone of P0 mouse CA1 region) was both low in volume and in density. Hence, total RNA was isolated from LCM-enriched samples pooled across ~6 slides per genotype.
14. After microdissection, carefully retrieve the collect tubes, close the cap upside down and vortex for 1 min.
15. Give the tubes a brief spin in the centrifuge in order to collect sample at the bottom of the tube. Store tubes at −80 °C for further analysis.
  Note: If more than one cell type or cell population is needed from one slide, collect one type of tissue from all sections at the same time and make sure that the cut tissue had been detached from the section before starting to collect others. Load the collection tube for the next cell/tissue type in a different hole to avoid possible tissue contamination.
16. Store unfinished PEN slides (where further LCM is possible) in the vacuum desiccator with desiccant at room temperature up to 5 days.

### G. RNA isolation

1. Pool all the collected RLT buffer (with micro-dissected samples of each cell/tissue type of each biological replicate) into a new, RNase-free 1.7 ml tube.
2. Vortex the tubes for 30 s.
3. Add an equal amount of 70% ethanol (RNase-free).
4. If the total volume is > 700 μl, transfer ~700 μl mixture each time to an RNeasy MinElute spin column and repeat this step using the same column, until the entire volume of mixture is used.
5. Follow the instruction given in the manual of RNeasy Micro Kit to isolate RNA from the LCM-ed samples.
6. Elute the RNA with 15 μl RNase-free water contained RNase Inhibitor (1 U/μl).
7. Repeat Step H6. If necessary, use the same tube for RNA collection.
8. Take 2 μl RNA elution into 2–3 μl RNase-free water for NanoDrop and RNA bioanalysis.
9. Proceed with measuring the concentration and purity of obtained RNA, as part of the data analysis.

Note: You may distribute each tube/genotype/sample into 3 technical replicates for further processing (for example, RNA sequencing).

## Data analysis

### Measure RNA concentration and RIN score

**Figure 5.**
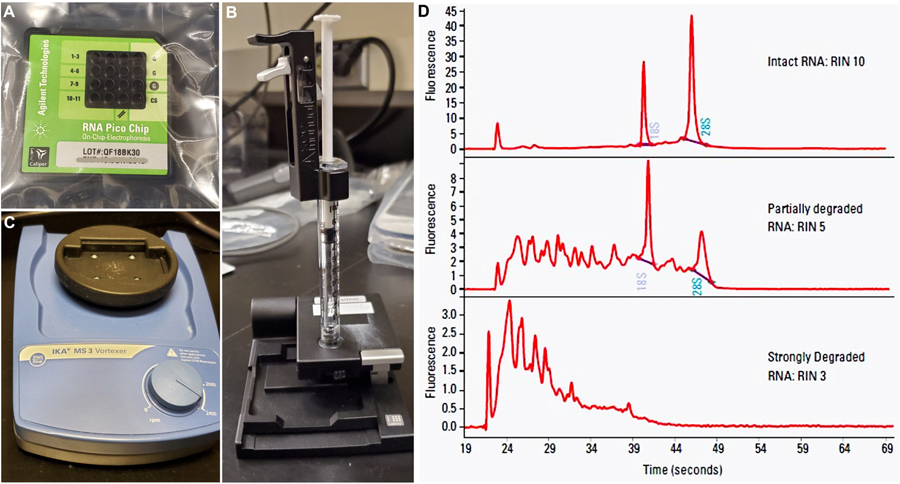
Components used to analyze isolated RNA. A. RNA Pico Chip, where the small quantity of RNA samples is loaded. B. Chip priming station, to be obtained separately to load the gel-dye mix into each sample-well of the Pico Chip. C. IKA vortex mixer is used to vortex the sample-loaded Chip before running it in the Agilent BioAnalyzer instrument. D. Representative image demonstrating different types of RNA integrity (modified from https://www.agilent.com/cs/library/applications/5989–1165EN.pdf).

1. After RNA is isolated by the RNAeasy kit, measure the concentration of RNA per area of interest, per genotype, per biological replicate, using the NanoDrop.
2. Calculate RNA quality by measuring the RNA Integrity Number (RIN) as well as RNA concentration (more precise than NanoDrop) for each RNA sample, using the Agilent Bioanalyzer 6000 Pico Kit.
  Note: RNA sample of RIN > 5.0 is considered usable (where maximum score can be 10.0).
3. Follow the manual available with the Agilent Kit for the consecutive steps. Briefly, (https://www.agilent.com/cs/library/usermanuals/Public/G2938–0049_RNA6000Pico_QSG.pdf):
  - Thaw RNA ladder on ice (avoid excessive warmth).
  - Prepare RNA gel matrix prior to the experiment (maximum: 4 weeks) and store at 4 °C.
  - Prepare the gel-dye mixture (components available within the kit) such that it is used within a day.
  - Load the gel-dye mixture carefully into each well of a new, sterile RNA Pico chip, with the help of the priming station (Figures 5A–5B).
  - Load RNA conditioning solution and RNA marker.
  - Load the thawed RNA ladder and samples. Vortex the Chip using the IKA vortex mixer (Figure 5C) before placing the former in the Agilent Bioanalyzer equipment for analysis.
  - Analyze RNA concentration and integrity using the Agilent software (Figure 5D).
4. With good quality of RNA thus obtained, proceed for different downstream analyses, as needed. These can include RNA sequencing (tissue, single cell), quantitative Real-Time polymerase chain reaction (RT-PCR), protein assays (Figure 4).

Note: In our experience, RNA concentration of 1.5–7 ng/µl and integrity of > 7.0 was sufficient to proceed to successful RNA sequencing (see Methods, Figure 4 and Figure supplements 1–3, Source files 1–2 in Roy et al., 2019).

## Recipes

Note: Use 100% RNase-free molecular grade water in the following recipes.

1. 70% ethanol (100 ml)
  - Add 70 ml RNase-free absolute (100%) ethanol and 30 ml water to make 100 ml of 70% ethanol
  - Store at −20 °C
2. 95% ethanol (100 ml)
  - Add 95 ml RNase-free 100% ethanol and 5 ml water to make 100 ml of 95% ethanol
  - Store at −20 °C
3. RNaseZap/70% ethanol (100 ml)
  - Mix RNaseZap and 70% ethanol (Recipe 1) in 1:1 ratio. For 100 ml, measure 50 ml of RNaseZap and 50 ml of 70% ethanol into a clean, RNase-free wash bottle
  - Mix the two liquids by rocking the bottle sideways for 4–5 min
4. 75% ethanol (freshly-prepared)
  - Add 75 ml RNase-free 100% ethanol and 25 ml water to make 100 ml of 75% ethanol
  - Store at −20 °C
5. Nissl stain for RNA research (50 ml)
  - Measure 0.5 g cresyl violet powder in a 50 ml RNase-free tube
  - Add 100% ethanol (RNase-free), final concentration: 1% solution (weight/volume)
  - Shake the solution for a few hours and store at 4 °C, sealed air-tight and dark for up to 6 months. This solution should be prepared at least 3 days prior to usage. Aliquot as required and discard the used solution.

## Footnotes

Ethics Animal experimentation: All animal experimentation was conducted in accordance with the guidelines laid down by the Institutional Animal Care and Use Committees (IACUC) of Seattle Children’s Research Institute, Seattle, WA, USA (protocol ID: IACUC00006; last day of triennial approval period: 11^th^ July 2021).
